# The Seasonality of Acute Attack of Primary Angle-Closure Glaucoma in Beijing, China

**DOI:** 10.1038/s41598-018-21074-w

**Published:** 2018-03-05

**Authors:** Jingyuan Zhu, Yang Xu, Hongyuan Wang, Dongjing Liu, Jingbo Zhu, Huijuan Wu

**Affiliations:** 1 Department of Ophthalmology, People’s Hospital, Peking University, 11 Xizhimen South Street, Xicheng District, Beijing, 100044 China; 2 Key Laboratory of Vision Loss and Restoration, Beijing Key Laboratory of Diagnosis and Therapy of Retinal and Choroid Diseases, Ministry of Education, 11 Xizhimen South Street, Xicheng District, Beijing, 100044 China; 3 Epidemiology and Biostatistics Department, Health Science Center, School of Public Health, Peking University, Beijing, 100191 China; 4 Middle School Attached to Beijing Jiaotong University, Beijing, China

## Abstract

In this study, the seasonality of acute attack of primary angle-closure glaucoma (PACG) was analysed. This retrospective case series included 283 patients (200 women, 83 men; mean age, 68.2 ± 10.3 years; range, 37–96 years) with acute attack of PACG from a university-based clinic over 4 years. Patients’ age and sex, and the date and season of onset of PACG attack, were analysed. Descriptive analysis and von Mises distribution were used for statistical analysis. The highest incidence of acute attack of PACG was observed in those aged 60–69 years (34.6%). Descriptive analysis showed that the incidence was greater in June and July for men, November for women, and November for the entire sample. An angular plot (using von Mises distribution) of the individual dates of onset revealed the estimated peak onset on September 11, November 8, and October 28 for men, women, and both, respectively. Integration of the results from the two analyses revealed the incidence to be higher in the summer and winter for men, and in the winter for women and for the entire sample. More females than males were affected. Monthly and seasonal variations in onset were observed, which might be related to weather changes.

## Introduction

Glaucoma is one of the leading causes of blindness worldwide^1^. Acute attack of primary angle-closure glaucoma (PACG) is an ophthalmologic emergency. Over the past decades, there have been few published epidemiological studies investigating the incidence and seasonality of acute attack of PACG in the general population, and an association, though with a low level of significance, has been reported between PACG attack and seasonal variations^2–4^. A study from Taiwan found a significant association between relative humidity and monthly admission rates for PACG among males, and for the population in the age range of 60 to 69 years^5^. A higher incidence was also noted during months with less sunlight (November, December, and January), due to mydriasis^6^. A statistically significant predominance of attacks occurring during summer and winter was observed in the Negev region of Israel^7^. In contrast, a study from southern Croatia demonstrated absence of a statistically significant association between the incidence of acute angle-closure glaucoma and seasonal variation^2^. Another study from Croatia also reported no seasonal variations in the occurrence of acute PACG and no correlation of the incidence of PACG with the mean duration of light exposure per season^3^. Nevertheless, in Croatia, the correlation between acute glaucoma and sunshine in November cannot be directly attributed to meteorological factors, although the amount of sunshine in different seasons (p < 0.01 in winter) and the incidence of acute angle-closure glaucoma seem to be inversely correlated^4^. So far, there have been no studies investigating this issue in mainland China. Hence, in the present retrospective study, we examined the relationship between the incidence of acute attack of PACG and seasonal variations, sex, and age at onset.

## Methods

The research protocol was approved by the ethics review board of the People’s Hospital of Peking University. The study procedures were performed in accordance with institutional guidelines and the declaration of Helsinki. Informed consent was obtained from all patients after providing them a full explanation of the procedures. The study was a retrospective chart review of 283 hospital outpatients treated for PACG at the Eye Centre of People’s Hospital of Peking University, Beijing, China, over a 4-year period from June 2011 through May 2015. The computer information system of this hospital is ranked second in Asia and first in China, and has cleared level 7 of the United States Healthcare Information and Management Systems Society (HIMSS). The fact that PACG necessitates either laser or filtration surgery and, therefore, has traceable records, enabled us to perform a study on the incidence of PACG with reliable and verifiable results.

PACG was diagnosed according to the International Classification of Diseases, Ninth Revision, Clinical Modification, code 365.2. The typical symptoms of acute attack of PACG are sharp vision loss and intense ocular pain, accompanied by systemic symptoms like severe forehead ache above the affected eye, and nausea and vomiting^8^. Cases of secondary glaucoma, open angle glaucoma, neovascular glaucoma, and readmissions were excluded from the analysis.

The case records of patients eligible for the present study were analysed and the following data were noted: age, sex, and month or season of the year when the acute attack occurred (in Beijing: spring, February to April; summer, May to July; autumn, August to October; and winter, November to January). The results were subjected to descriptive and graphical analyses, and von Mises distribution^9,10^ was used to calculate and determine the significance of the results. Because the distribution of onset dates is on a circle, rather than along a line, the use of the normal distribution to describe their distribution is not appropriate, and the von Mises distribution has been proposed to describe seasonal data with a single peak^9^. This distribution was termed the “natural” analogue of the normal distribution for seasonal data with a single peak^10^. Accordingly, we used the von Mises distribution for seasonal analysis.

## Results

### Patient Age

**Figure 1:**
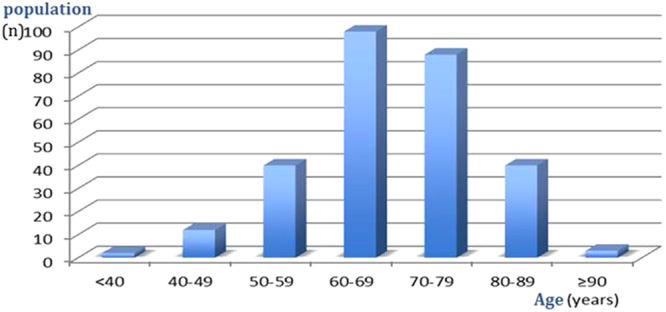
Age distribution of attack of primary angle-closure glaucoma (PACG) in the entire cohort. The histogram shows the increasing number of individuals who experienced attack of acute PACG in the 60- to 69-year age group, compared with other age groups.

A total of 283 patients were included in the analysis, including 200 women (70.7%) and 83 men (29.3%), corresponding to a female to male ratio of 2.4:1. The age at diagnosis ranged from 37 to 96 years (mean ± SD, 68.2 ± 10.3 years). The highest incidence of acute attack of PACG (34.6%) was observed in both sexes in the age range of 60 to 69 years (Table 1, Fig. 1).

**Table 1:** Age distribution of attack of primary angle-closure glaucoma (PACG) from year 2011 to 2015.

Age (years)	n
<40	2
40–49	12
50–59	40
60–69	98
70–79	88
80–89	40
≥90	3

*n: numbers of people*.

### Monthly variation in incidence

**Figure 2:**
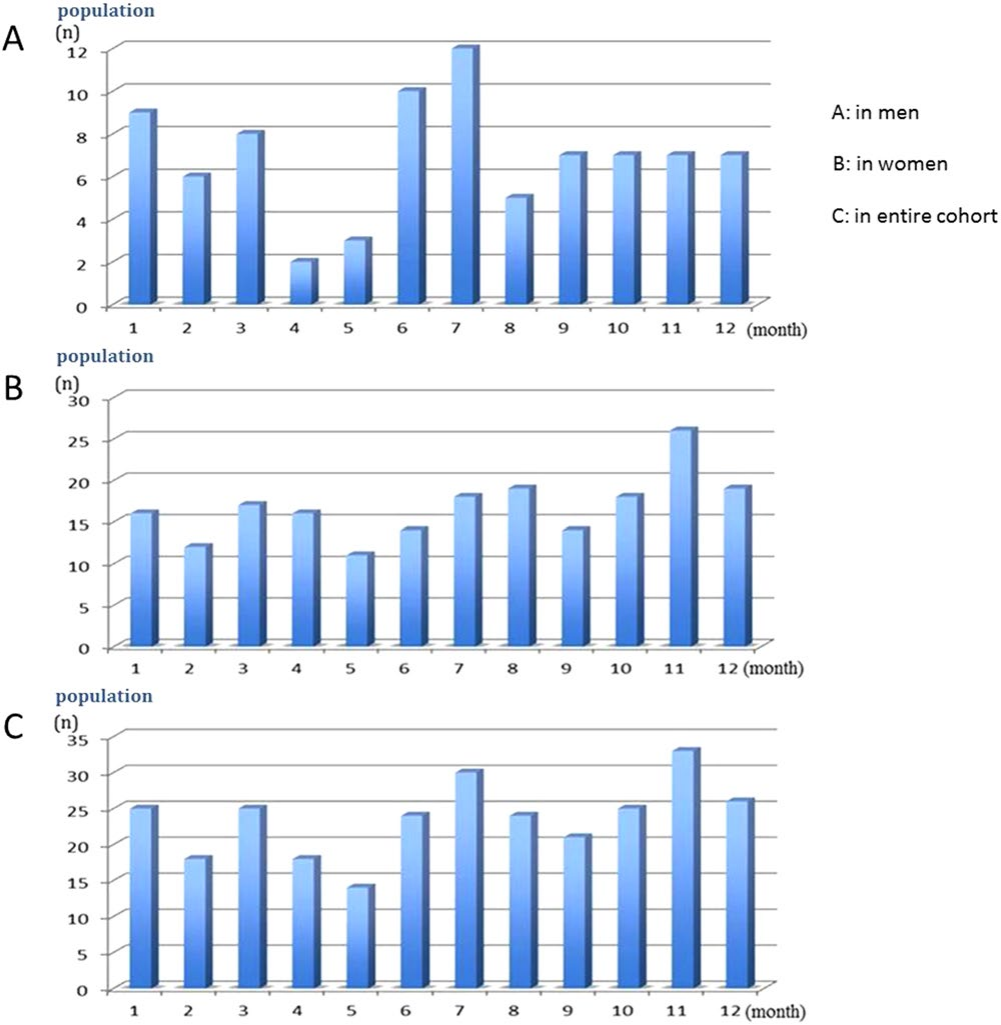
Monthly distribution of attack of primary angle-closure glaucoma (PACG). (**A**) The histogram reveals changes in the distribution of male patients with acute PACG, and the greater numbers in June and July. (**B**) The histogram reveals changes in the distribution of female patients with acute PACG, and the greater numbers in November. (**C**) The histogram reveals changes in the distribution of entire cohort with acute PACG, and the greater numbers in November.

Descriptive analysis: The incidence of acute attack of PACG was highest in June and July for men, November for women, and November for the combined sample (Table 2, Fig. 2A–C).

**Table 2:** Monthly distribution of attack of primary angle-closure glaucoma (PACG) according to sex.

Month	Male, n (%)	Female, n (%)	Total, n (%)
January	9 (10.8)	16 (8.0)	25 (8.8)
February	6 (7.2)	12 (6.0)	18 (6.4)
March	8 (9.6)	17 (8.5)	25 (8.8)
April	2 (2.4)	16 (8.0)	18 (6.4)
May	3 (3.6)	11 (5.5)	14 (4.9)
June	10 (12.0)	14 (7.0)	24 (8.5)
July	12 (14.5)	18 (9.0)	30 (10.6)
August	5 (6.0)	19 (9.5)	24 (8.5)
September	7 (8.4)	14 (7.0)	21 (7.4)
October	7 (8.4)	18 (9.0)	25 (8.8)
November	7 (8.4)	26 (13.0)	33 (11.7)
December	7 (8.4)	19 (9.5)	26 (9.2)
**Total**	83 (100)	200 (100)	283 (100)

Peak periods: June and July for men; November for women; November for both sexes.

**Figure 3:**
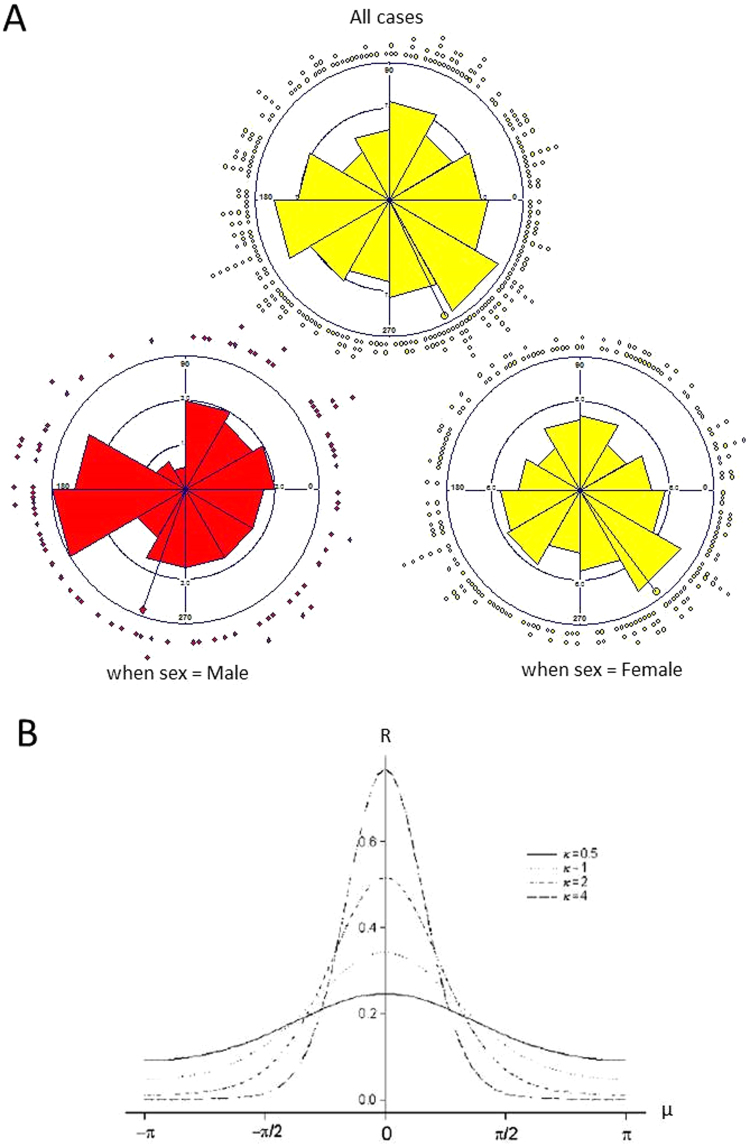
Angular plot (rose plot) of the dates of onset of primary angle-closure glaucoma (PACG), and probability density functions of the von Mises distribution. (**A**) The 360 angles correspond to dates of the 365 days in a year. The segments are ordered from January to December corresponding from 0°to 360° (anticlockwise) starting due east in each rose plot. Corresponding peak onset and magnitude of each rose plot is indicated by the arrow analogues, which point to different angles (Above: all cases; left below: male data; right below: female data). (**B**) R is the magnitude of the peak estimated by the length of the mean resultant; μ stands for mean direction. The functions with μ = 0°, for κ = 0.5, 1, 2 and 4, were used to better understand the shape changes in the application of von Mises distribution^9^.

In the application of the von Mises distribution, data from each calendar year are standardized to 365 days and then converted to an angle between 0° and 360°. We illustrated the data on attack of PACG graphically in a rose diagram format (angular plot, Fig. 3A), where each petal represented a ‘standard’ month or an angle of 30° (360°/12). In Fig. 3B, R is the magnitude of the peak estimated by the length of the mean resultant, and μ stands for mean direction. As κ → 0, the distribution tends to become uniform. The larger the value of κ, the greater is the clustering around the mode^9^. The shapes of the corresponding (μ; κ) with μ = 0° for various values of κ are shown.

Graphical analysis and von Mises distribution: An angular plot of the 283 individual dates of onset of PACG is shown in Fig. 3A, and the corresponding data are presented in Table 3. For the combined sample (i.e. including both men and women), the estimated peak of onset was on October 28 (95% confidence interval [CI], August 28 to December 28), with μ_0_ = 296.15°. In men, the estimated peak of onset was on September 11, with μ_0_ = 250.40°. In women, the estimated peak of onset was on November 8 (95%CI, August 25 to January 22), with μ_0_ = 307.10°. All three of these peaks were statistically significant (U2_Watson_ = 57.439 and p = 0.005 for the combined group; U2_Watson_ = 13.458 and p = 0.005 for men; U2_Watson_ = 43.635 and p = 0.005 for women)^11^, but were of modest magnitude (R = 0.08 and κ = 0.17 for the combined group; R = 0.06 and κ = 0.13 for men; R = 0.10 and κ = 0.20 for women).

**Table 3:** Estimated peak date of onset of acute attack of primary angle-closure glaucoma (PACG) for all patients and according to sex.

	n	R	κ	Peak (μ_0_°)	Date of peak	Difference (days)	95% Bootstrap	U2 (Watson)	P
Total	283	0.08	0.17	296.15	Oct 28		Aug 28–Dec 28	57.439	0.005
Sex									
Male	83	0.06	0.13	250.40	Sep 11	—	—	13.458	0.005
Female	200	0.10	0.20	307.10	Nov 8	58	Aug 25– Jan 22	43.635	0.005
Age, years									
37–49	14	0.18	0.00	41.70	Feb 12	—	—	3.278	0.005
50–59	40	0.15	0.31	275.93	Oct 7	237	Jul 6– Jan 8	5.045	0.005
60–69	98	0.10	0.20	332.41	Dec 4	295	Sep 3– Mar 5	23.381	0.005
70–79	88	0.09	0.17	228.05	Aug 20	189	Apr 21– Dec 19	9.561	0.005
80–96	43	0.18	0.36	303.34	Nov 4	265	Aug 21– Jan 18	8.874	0.005

^°^Angle in degrees. Apr, April; Aug, August; Dec December; Feb, February; Jan, January; Mar March; Nov, November; Oct, October; Sep September.

### Seasonal variation in incidence

**Figure 4:**
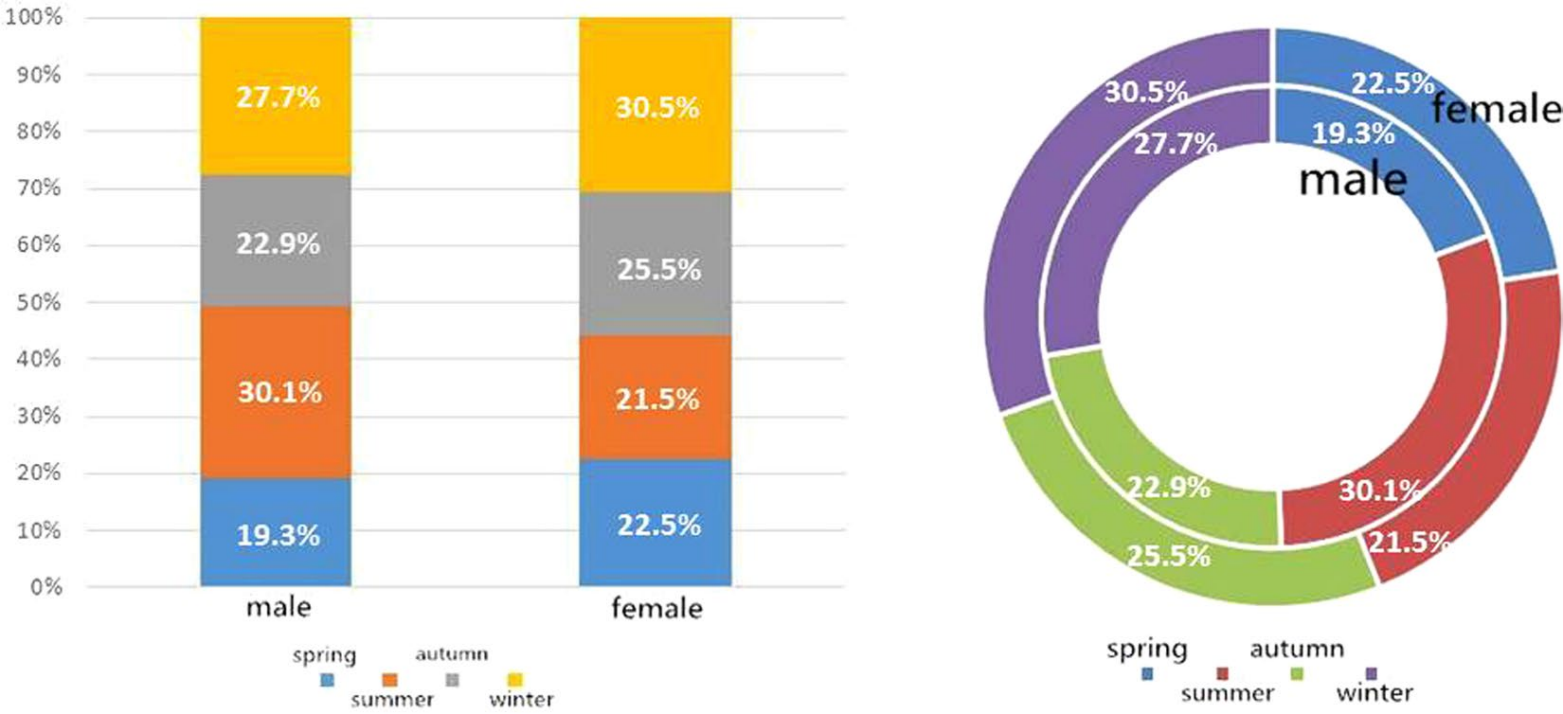
Seasonal distribution of attack of primary angle-closure glaucoma (PACG). The left bar chart represents the incidence of acute attack of PACG in different sexes and different seasons. The right dount chart better demonstrates the proportion of the incidence of acute PACG in different sexes and different seasons. Both show a greater number in the summer and winter for men, winter for women, and winter for both sexes.

In Beijing, the spring season spans from February to April, summer from May to July, autumn from August to October, and winter from November to January. The data on seasonal variations in the number of PACG outpatients for each sex, and for the combined sample are summarised in Table 4. In the descriptive analysis, the incidence of acute attack of PACG over the 4 years was highest in the summer and winter for men, and in winter for women as well as for the combined group (Table 4, Fig. 4).

**Table 4:** Seasonal distribution of attack of primary angle-closure glaucoma (PACG) according to sex.

Season	Male, n (%)	Female, n (%)	Total, n (%)
Spring	16 (19.3)	45 (22.5)	61 (21.6)
Summer	25 (30.1)	43 (21.5)	68 (24.0)
Autumn	19 (22.9)	51 (25.5)	70 (24.7)
Winter	23 (27.7)	61 (30.5)	84 (29.7)
**Total**	83 (100)	200 (100)	283 (100)

Peak periods: Summer *a*nd winter for men; winter for women; and winter for both sexes.

According to the results of the von Mises distribution, the highest incidence of acute attack of PACG for females (October 28) and for the entire sample (November 8) appears to be in the winter (around November). Thus, there were statistically significant (p = 0.005, Table 3) seasonal variations in the occurrence of acute attack of PACG.

## Discussion

In this retrospective study, we investigated the seasonality of acute attack of PACG in outpatients treated at a hospital in Beijing. The observation that acute attack of PACG affects women more frequently than men^12^ has been reported previously, and is in line with the results of our study; we found that acute attack of PACG affected women more than twice as often as men. Various authors have reported the highest risk for acute attack of PACG to be around the seventh decade (60 to 69 years of age) of life^5–7^. In our study, the highest incidence was observed in the same age group. The prevalence of acute attack of PACG has been found to increase proportionately with age for different racial groups^12^. This age-related increase may be due to the anterior chamber becoming shallower with increasing age^13^. Other possible reasons for increasing predisposition to an acute attack of PACG include the following: use of mydriatic drugs^6,14^; low background illumination^14^; emotional disturbances (antidepressant use)^15,16^; increased antero-posterior lens diameter due to prolonged reading^6^; performing sustained Yoga postures^17^; meteorological factors such as sunspot activity^18^, and increased relative humidity^5^. A previous study from Romania reported the following: “the increase of antero-posterior lens diameter (intumescent lens) is just a starting factor for a potential primary closed-angle glaucoma”^6^.

However, from the circular plot in our study, it is clear that there are some evidences of more than a single mode which causing acute attack of PACG, but these evidences do not appear to be strongly related to, for example, sex (Table 3); the peak onset for women occurred 58 days later than that for men. Nevertheless, the von Mises distribution provides a reasonable description of the data, with a stronger peak onset of PACG indicated for different age group with p = 0.005 (Table 3). Hence, there were statistically significant monthly and age-dependent variations in the incidence of acute attack of PACG.

There are limited data in the existing literature regarding the seasonality of acute attack of PACG in mainland China, though similar studies have been conducted in other parts of the world. November and December have been described as the months of the peak incidence in Finland^19^. In the United Kingdom also, an increased incidence has been reported in the winter months, especially in December^18^. While in Singapore, the incidence was found to be higher on hotter days (i.e., days with a higher number of attacks appeared to be hotter and drier, with more direct sunshine)^20^. A study by David *et al*. also found that in Israel, a significant number of attacks occurred during the summer and winter^7^. In our study, we observed a significant predominance of attacks during the summer and winter (for men) and during the winter (for women and for the combined sample), which is in line with the observation of a higher incidence of acute attacks of PACG coinciding with periods of extreme temperatures in the above-mentioned regions.

Meteorological conditions have previously been reported to be associated with the attack of PACG, and early significant results were only considered to be associated with shortened duration of sunshine. In Finland, an increase in the incidence of acute angle closure glaucoma was noted whenever the number of hours without sunshine increased^21^. Teikari *et al*. reported that the incidence of acute attack of PACG was higher in winter and autumn compared with spring and summer^21^. However, in Taiwan, hospital admission rates of PACG were significantly higher in March and with increased relative humidity^5^. Therefore, we believe that cold weather, especially in case of women, and hot dry summers especially in case of men, tend to drive everyone^22^, especially the elderly^23^, indoors (where poorer light acts as a surrogate for the darkroom test and makes patients susceptible to glaucoma).

In our study, we have investigated the difference between males and females in mainland China while previous studies were conducted on a combined sample and did not look into sex-related differences in the seasonality of acute attack of PACG. We used more advanced and robust statistical methods to analyse the trends in the incidence of acute attack of PACG, in contrast to previous studies, which used only the chi-square test; angular analysis based on von Mises distribution is more appropriate for seasonal studies^8,9^. This is a major strength of our study over previous studies. However, in our study, results on the peak onset in males obtained by descriptive analysis (June and July) were different from those obtained by Von Mises analysis (September 9th); therefore, the association between seasonal variations and acute attack of PACG is not as clear in men as that in women. Thus, we acknowledge that our statistical methods may need further improvement. Better methods for detecting and treating this disease are still needed, as is optimization of the appropriate statistical approach. In conclusion, our results indicate that seasonal variations during the year seem to be an important factor affecting the occurrence of acute attack of PACG. Findings from the present study will contribute to an improved understanding of the epidemiology of acute attack of PACG.
